# Endocannabinoid System in the Airways

**DOI:** 10.3390/molecules24244626

**Published:** 2019-12-17

**Authors:** Turgut Emrah Bozkurt

**Affiliations:** Department of Pharmacology, Faculty of Pharmacy, Hacettepe University, Ankara 06100, Turkey; turgutb@hacettepe.edu.tr; Tel.: +90-533-226-8494 or +90-312-305-2131

**Keywords:** cannabinoids, endocannabinoids, airway, cannabinoid receptor, fatty acid amide hydrolase, monoacylglycerol lipase

## Abstract

Cannabinoids and the mammalian endocannabinoid system is an important research area of interest and attracted many researchers because of their widespread biological effects. The significant immune-modulatory role of cannabinoids has suggested their therapeutic use in several inflammatory conditions. Airways are prone to environmental irritants and stimulants, and increased inflammation is an important process in most of the respiratory diseases. Therefore, the main strategies for treating airway diseases are suppression of inflammation and producing bronchodilation. The ability of cannabinoids to induce bronchodilation and modify inflammation indicates their importance for airway physiology and pathologies. In this review, the contribution of cannabinoids and the endocannabinoid system in the airways are discussed, and the existing data for their therapeutic use in airway diseases are presented.

## 1. Cannabinoids and the Endocannabinoid System

The plant cannabis sativa, produces a variety of biologically active cannabinoids and related compounds. ∆^9^-tetrahydrocannabinol (THC) is the predominant molecule with 1–10% by weight. It was first extracted in 1964 by Gaoni and Mechoulum and is now known to be the primary molecule responsible for most of the biological effects of the cannabis plant [1]. Thereafter, in 1992, *N*-arachidonylethanolamine “anandamide” (AEA) was isolated from swine brains, which was the first endogenous cannabinoid related substance isolated from a mammal [2]. Related substances were also isolated from gastrointestinal tissues [3,4]. In the following years, multiple endogenous cannabinoid molecules like 2-arachidonylglycerol (2-AG), noladin ether, virodhamine, and oleoyl ethanolamine were identified and these substances were then named as “endocannabinoids”, which are derivatives of arachidonic acid conjugated with ethanolamine or glycerol. Endocannabinoids, their receptors, and metabolic pathways form the “Endocannabinoid System”, a term which was first used by Di Marzo and Fontana [5].

Among the wide variety of endocannabinoids, AEA and 2-AG have attracted a significant number of researchers in the endocannabinoid area. AEA is synthesized by the enzymes *N*-acyl-phosphatidylethanolamine phospholipase D (NAPE-PLD), α/β-hydrolase-4 (Abh4), and phospholipase-C (PLC)-catalyzed cleavage of NAPE to phosphoanandamide and then to AEA [6,7]. It is mainly catabolized by fatty acid amide hydrolase (FAAH), the enzyme which also catabolizes the non-cannabinoid fatty acids [8]. In addition to FAAH, *N*-acylethanolamine acid amidase (NAAA) has also been identified as another hydrolase for AEA [9]. 2-AG is synthesized by phospholipase C (PLC) and diacyl-glycerol-lipase (DAGL), and catabolized mainly by monoacylglycerol-lipase (MAGL) [10]. Although MAGL is the predominant enzyme for 2-AG metabolism, α/β-hydrolase-6 (Abh6) and α/β-hydrolase-12 (Abh12) also contribute to a minor degree. FAAH has a negligible effect in 2-AG catabolism [7]. In macrophages, it was also shown that carboxylesterase-1 (CES1) and palmitoyl protein thioesterase-1 (PPT1) could hydrolyze 2-AG [11,12]. Nevertheless, cyclooxygenase-2 (COX-2) is involved in the oxidation of both 2-AG and AEA [13]. COX-2 oxidizes AEA to generate prostamides, like prostaglandin H_2_ ethanolamide (PGH_2_-EA) and prostaglandin H_2_ (PGH_2_), respectively [14], and 2-AG to prostaglandin H_2_ glycerol (PGH_2_-G) [14,15].

Cannabinoids exert their biological effects mainly through two 7-transmembrane (TM) receptors, namely cannabinoid receptor CB_1_ and CB_2_ [16]. However, they can also bind to other targets like transient receptor potential vanilloid receptor1 (TRPV1) [7,17], orphan receptors G protein-coupled receptor-55 (GPR55) [18,19], GPR18 [20], GPR110 [21], GPR119 [22], and peroxisome proliferator-activated receptors (PPARs) (mostly PPARα) [22]. The cannabinoid receptors are G-protein coupled receptors (GPCRs) (G_i/o_) and human CB_1_/CB_2_ receptors share 44% overall homology [16,23]. Since mice have been used in several cannabinoid related studies, it is also important to note that human and mouse CB_1_ receptors share 96% [24], and CB_2_ receptors share 82% homology [25], where mouse CB_1_ and CB_2_ receptors share 66% homology [25]. Both CB_1_ and CB_2_ receptors are negatively coupled to adenylyl cyclase and stimulate mitogen-activated protein kinase (MAPK). Furthermore, they also activate K^+^ channels and inhibit Ca^++^ channels, both of which result in the inhibition of transmitter release via the activation of G_β/γ_ subunit [26,27,28].

CB_1_ receptors are mainly located in the nervous system, nerve terminals and a wide range of tissues including adipose tissue, liver, gastrointestinal tract, whereas CB_2_ receptors are expressed in peripheral tissues, primarily in immune cells, immune-related organs and tissues like tonsils, spleen, thymus and bone marrow [7,29,30]. However, similar to CB_1_, CB_2_ receptor expression has also been demonstrated in various tissues and cells such as the brain, spinal cord [31,32], lung, testes [23,33], osteoblasts, osteocytes, and osteoclasts [34].

Although most of the research has been focused on the central nervous system effects of cannabinoids, they have various biological effects in the immune, cardiovascular, gastrointestinal, and respiratory system [35]. These effects had attracted the interest in the clinical use of cannabinoids. However, most of the cannabinoid related drugs have been withdrawn from the market due to unacceptable central side effects, which are mainly mediated by CB_1_ receptors. Today “Sativex^®^” is the only cannabinoid-containing product, which consists of ∆^9^-tetrahydrocannabinol (THC) and cannabidiol, and have been approved for the spasticity of multiple sclerosis.

Cannabinoids have a significant contribution to the immune modulation [36], which suggests their importance in pathophysiological conditions and inflammatory diseases. The alteration of endocannabinoid levels by inflammation was observed in several studies performed in the brain, liver, coronary artery, and colon [37,38,39,40,41]. Furthermore, endocannabinoids and activation of the cannabinoid system have also been demonstrated to inhibit inflammatory responses in hepatitis [42], pulmonary inflammation [43], inflammatory pain [44], sepsis [45] and colitis [46] by reducing the recruitment of inflammatory cells and increasing the production of anti-inflammatory cytokines. Earlier studies have indicated that cannabinoids can inhibit antibody production [47,48]. THC has been shown to suppress Th1 and enhance Th2 cytokines, and AEA has been demonstrated to decrease T and B cell proliferation [49,50]. However, the immune-modulatory effects of cannabinoids can also lead to the down-regulation of immune responses, which can cause the augmentation of infections and tumorigenesis [51,52,53].

The intense involvement of cannabinoids in immunomodulation also suggested the role of CB_2_ receptors as they are highly expressed in the immune system. CB_2_ receptors are involved in B-cell differentiation [54], macrophage migration [55], and antigen processing [56]. CB_2_ receptors also contribute to mast cell activation by generating nitric oxide (NO) and prostaglandin-E_2_ (PGE_2_) [57]. Besides CB_2_ receptors, the existence of functional CB_1_ receptors was also shown in mast cells [58], suggesting both receptors are also important in allergic diseases [59,60]. On the other hand, when used in higher concentrations, the effects of cannabinoids on inflammation can occur through cannabinoid receptor-independent mechanisms such as PPARs [61,62].

## 2. The Role of Endocannabinoid System in the Airways

It is known that marijuana smoking can cause cellular damage in the lungs. However, there are some differences in the airways of marijuana and tobacco smokers [63,64]. Gong et al. have shown that both marijuana and tobacco smokers have goblet and basal cell hyperplasia with a tendency of higher hyperplasia in the marijuana group [65]. There was also cellular disorganization in more than 50% of marijuana smokers. In a study performed in primates, smoking marijuana was shown to induce bronchiolitis, alveolar cell hyperplasia, and fibrosis in greater incidence when compared to that of the cigarette group [66]. Therefore, it can be concluded that the plant-derived cannabinoids may have significant effects in the airways.

**Figure 1:**
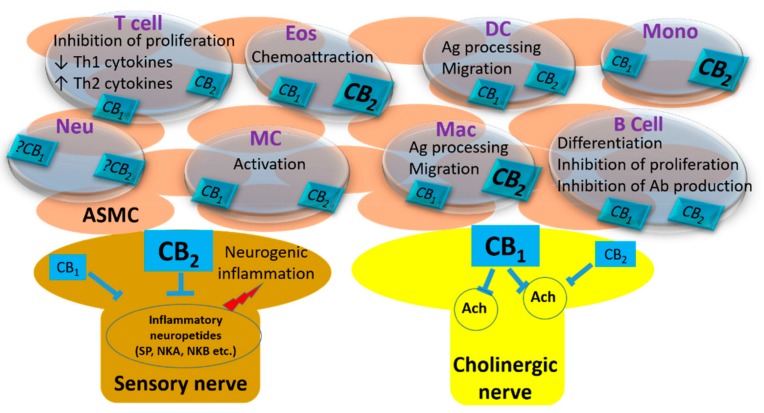
The localization CB receptors and their reported effects in the airways. (ASMC: Airway smooth muscle cell; Eos: Eosinophil; DC: Dendritic cell; Mono: Monocyte; Neu: Neutrophil; MC: Mast cell; Mac: Macrophage; Ag: Antigen; Ab: Antibody). The receptors in bold characters represent a denser expression in that cell type.

The synthesis of endocannabinoids in the airways is established in various cell types (Figure 1). Although the data about the expression of CB_1_ and CB_2_ cannabinoid receptors in human airways are not clear, it is known that they are mostly expressed by the immune cells within the airways [67,68]. CB_2_ receptors are densely expressed on eosinophils [69,70,71] and monocytes, both of which also express CB_1_ receptors [33,72,73,74]. Eosinophil recruitment to the airways is an important process in the chronic inflammatory state of allergic asthma. The significant amount of CB_2_ receptors in human eosinophils indicates a critical response capacity of these cells to cannabinoids [75]. In this regard, 2-AG was shown to be a chemoattractant factor for human primary eosinophils [69,76,77]. Moreover, interleukin-5 (IL-5), which is an important mediator for eosinophil differentiation and priming, can enhance the effect of 2-AG [77]. Selective stimulation of CB_2_ receptors with synthetic ligands is also able to modify the activity of eosinophils to chemoattractants [78]. These studies indicate that endocannabinoids have a significant contribution to eosinophil recruitment. Human monocyte-derived dendritic cells (DCs) isolated from peripheral blood and murine bone marrow-derived DCs express both CB_1_ and CB_2_ receptors, which can synthesize AEA and 2-AG [49,79,80]. Studies have shown that the activation of DCs by cannabinoid ligands can inhibit the release of inflammatory cytokines [49] and can suppress the immune response by inducing apoptosis of these cells [79]. The interaction of DCs with T cells should also be considered for the effects of cannabinoids in the immune response. Do et al. suggested that inflammation-induced 2-AG production by DCs can affect cannabinoid receptors on T cells and switch the immune response from Th2 to Th1, and T cells can produce endocannabinoids which may affect their receptors on DCs. [80]. The expression of cannabinoid receptors and their function is more complicated in neutrophils. Detectable CB_2_ mRNA levels were shown in polymorphonuclear neutrophil cells [33]. However, Chouinard et al. have demonstrated that human neutrophils do not express a significant amount of CB_2_ receptor protein, although they are responsive to selective agonists [70,71,81].

### 2.1. Airway Reactivity

In 1973, Tashkin et al., have performed a study in healthy volunteers, in which they have investigated the effects of cannabinoids in the airways. Interestingly, marihuana inhalation and oral ingestion of THC had produced bronchodilation lasting for sixty minutes and six hours, respectively [82]. In the meantime, Vachon et al. have demonstrated a similar bronchodilatory effect by cannabinoids in healthy volunteers and have shown that the effect was dose-dependent [83]. Furthermore, in the doses that they produce bronchodilation, cannabinoids did not cause central respiratory depression.

These findings suggested the role of cannabinoids in asthma treatment. In two different studies, Tashkin et al., have shown that smoking marijuana or ingestion of THC by subjects with chronic, clinically stable, bronchial asthma of minimal or moderate severity, can produce bronchodilation [84,85]. A few years later, Hartley et al. have demonstrated that the bronchodilator effect of THC can be observed in concentrations that do not cause central or cardiovascular effects [86]. However, the mechanism for the bronchodilatory effect of cannabinoids was not known, and this effect did not appear to be related to β-adrenoceptor stimulation or cholinergic blockade [86].

The physiological significance of these studies and their therapeutic potential were complicated because some asthmatic patients had responded to these compounds with paradoxical bronchospasm [87,88]. In 2000, Calignano et al. have performed a study in rats and guinea pigs in order to investigate this controversy [89]. They have demonstrated that cannabinoids have bidirectional effects in the airways, depending on the airway tone. In their study, AEA had inhibited bronchial responsiveness to chemical irritation in rodents but caused bronchospasm when the constricting tone was removed. They have shown that both effects were mediated through peripheral CB_1_ cannabinoid receptors located on axon terminals of airway nerves [89].

In the isolated organ bath experiments, AEA and synthetic CB_2_ receptor agonist WIN 55,212-2 was shown to inhibit electrical field stimulation (EFS)-induced contractions of the rat tracheal rings by acting pre-junctionally [90]. This inhibitory effect was no surprise considering the inhibitory second messenger pathways mediating the effects of CB_1_ and CB_2_ receptors. In accordance with this data, cannabinoid agonists have been shown to inhibit acetylcholine release from cholinergic nerves via activation of CB_2_ [91]. However, this inhibition was not associated with a functional response.

Research on the effect of cannabinoids in the airways was mostly focused on airway inflammation after that time, and no new data was produced about their functional roles until the work by Grassin-Delyle et al. was published [92]. They have shown that pre-junctional CB_1_ receptor activation mediates the inhibition of cholinergic nerve mediated contractions in the human bronchus. Therefore, they have suggested this mechanism for the explanation of acute bronchodilation produced by marijuana smoking. Bozkurt et al. have also demonstrated that CB_1_ stimulation can inhibit the increased neuronal activity and nerve density in airway inflammation and can directly inhibit cholinergic contractions by a presynaptic mechanism, indicating a protective role of CB_1_ receptors in airway inflammation [93]. These findings indicate that cannabinoids can inhibit bronchoconstriction by a pre-junctional inhibition of neurotransmission.

### 2.2. Airway Diseases

Allergen challenge of asthmatic patients has been shown to result in increased AEA concentrations in their bronchoalveolar lavage (BAL) fluid [68]. This finding suggests the contribution of the endocannabinoid system in the pathophysiology of allergic asthma. It is also the first study to report the involvement of endocannabinoids in human asthma [68]. In that study, Zoerner et al. have demonstrated that allergen exposure increases AEA levels from about 5 pmol/L to 30 pmol/L in human BAL samples [68]. However, it cannot be concluded still, if the increased AEA concentrations is a cause or consequence of the pathophysiology of asthma in humans. As highlighted above, cannabinoids have significant anti-inflammatory effects; however, the endocannabinoid system can induce both pro-inflammatory and anti-inflammatory responses [94,95]. This controversy may be due to their heterogeneous targets other than cannabinoid receptors, like TRPV1, GPR119, GPR55, and PPARs [6]. However, in a study performed cardiomyocytes it has been shown that AEA concentrations should be around the micromolar range in order to induce tissue damage, which is very high when compared to that of normal tissue levels [95]. Therefore, as speculated by Zoerner et al., it can be concluded that the increase in AEA production could be a protective mechanism rather than a pathological component. AEA levels in the airways were reported as around 2 ng/mg tissue for mouse [96] and 0.3 pg/mL for rabbit lung [97], whereas 2-AG levels were about 20 ng/mg in the mouse lung [96].

Another cannabinoid related endogenous lipid mediator palmitoylethanolamide (PEA), has been shown to be decreased in the airways after allergen sensitization [98]. The concentration of PEA was reported to be around 6 pmol/mg in mouse harvested bronchi, which was reduced to 1 pmol/mg after ovalbumin sensitization [98]. PEA is co-released with AEA and behaves as a local autacoid down-regulator of mast cell activation and inflammation [98]. The supplementation of PEA has also been proposed to prevent the development of asthma-like features in the same study.

The plant-derived non-psychotropic cannabinoid cannabidiol, has immunosuppressive and anti-inflammatory effects [99]. In mice, cannabidiol has been shown to suppress lipopolysaccharide (LPS)-induced TNF-α production [100]. Ribeiro et al., have demonstrated in two different studies that cannabidiol can decrease inflammation and improve lung functions in LPS-induced acute lung injury in mice [101,102], which is further confirmed by Vuolo et al. [103]. The influence of cannabidiol on the antigen-induced contraction of guinea-pig airways has also been demonstrated [104]. However, it should also be noted that cannabidiol can cause drug interactions, hepatic abnormalities, diarrhea, fatigue, vomiting, and somnolence [105]. Therefore, these possible side effects and their severity should be considered for using cannabidiol as a therapeutic tool.

Studies have shown that endocannabinoids and cannabinoid CB_1_ receptors may have a significant inhibitory role in human mast cell degranulation and activation in the airway mucosa and skin, suggesting the contribution of the endocannabinoid system in the allergic diseases [59,60]. Martin-Fontecha et al. have shown that the expression of CB_1_ receptor has been upregulated in tonsils and peripheral blood of patients with allergic rhinitis, atopic dermatitis, and food allergy [106]. In accordance with these studies, the high expression of the CB_1_ receptor proteins were also demonstrated in B cells, T cells, pDCs, and mDCs of atopic donors [106]. Symptomatic allergic donors were also found to have higher expression of CB_2_ receptors on their eosinophils [78]. Frei et al. have shown that migratory responses of human and mouse eosinophils can be enhanced by selective activation of CB_2_ receptors through Gα_q_/MEK/ROCK (Gα_q_/ mitogen/extracellular signal-regulated kinase / rho-associated protein kinase) signaling [78].

The expression levels of cannabinoid receptors in the lungs were also shown to be affected by viral infections [107]. Tahamtan et al., have suggested in their studies that respiratory syncytial virus (RSV) infection of airways lead to an induction in CB_1_ receptor expression. However, in another study, they have also demonstrated the contribution of CB_2_ receptors for RSV infection in both mice and humans [108].

## 3. Targeting the Endocannabinoid System for the Treatment of Airway Diseases

Several pathological conditions have been associated with a change in the expression of cannabinoid receptors, altered endocannabinoid tissue concentrations, or a change in their metabolism. Therefore, different therapeutic strategies were considered by modifying the endocannabinoid system, such as targeting CB_1_/CB_2_ receptors or interfering with their metabolism [6].

Due to the immunomodulatory effect of cannabinoids, a significant variety of studies were focused on their possible therapeutic potential on inflammatory diseases like asthma [43,67,68,92,93,104,109,110,111,112,113,114]. The molecular mechanisms mediating the effects of cannabinoids in allergic airway responses mainly depend on their effects on immune cells and the related release of cytokines [115]. In mice, treatment with the plant-derived cannabinoids, cannabinol and THC, was able to inhibit the expression of critical T cell cytokines and inflammatory response in ovalbumin-induced experimental allergic airway inflammation [116]. Using the ovalbumin model, Braun et al., have demonstrated that THC can inhibit cell proliferation, suppress cytokine and chemokine production, and stimulate regulatory T cells [112]. However, they have also suggested that these effects are probably mediated by cannabinoid receptor-independent mechanisms [112].

Giannini et al. demonstrated that the non-selective cannabinoid receptor agonist CP-55,940 can prevent allergen-induced bronchospasm, and reduce cough and leukocyte recruitment in the lung [67]. This effect could be blocked by both CB_1_ and CB_2_ receptor blockade, suggesting that both receptors were involved in this effect [67]. In guinea pig airways, the synthetic cannabinoid ligand WIN-55212-2 has demonstrated a reduction in airway neurogenic inflammation in vivo [91]. This was due to an inhibition of C-fiber nerve activity and mediated by the activation of CB_2_ receptors. WIN-55,212-2 was also shown to inhibit airway plasma extravasation and bronchoconstriction induced by intra-esophageal HCl instillation in guinea-pigs, an effect which was also mediated through CB_2_ activation [113]. CB_2_ receptor modulation of airway sensory nerve activity and related cough reflex have been highlighted in several different studies [110,117]. These findings suggest that CB_2_ receptors can contribute to both neurogenic airway inflammation and hyperreactivity as well. This may also be related to a species difference since studies underscoring the CB_2_ receptor-mediated reactivity changes in the airways are mostly performed in guinea pigs. However, the crucial role of CB_2_ activation in the regulation of pulmonary natural killer (NK) cell function has also been demonstrated in mice [118]. Therefore, blocking the CB_2_ receptors in allergic airway inflammation may modulate NK cell response during airway inflammation. The plant-derived cannabinoid, cannabidiol, was also shown to inhibit allergen-induced contraction of airway smooth muscle, and reduce antigen-induced airway obstruction in guinea pigs [104]. This effect seems to be partly mediated by the inhibition of mast cell degranulation.

In the experimental studies performed in mice, selective CB_1_ receptor agonists have been shown to inhibit inflammation-induced hyperreactivity in an in vitro model of nerve growth factor (NGF)-induced neurogenic inflammation [93]. The inhibitory effect of CB_1_ receptors was demonstrated using two different synthetic selective CB_1_ agonists ACEA and ACPA. In the same study, these selective ligands were also able to inhibit nerve mediated cholinergic contractions, further confirming the inhibitory role of CB_1_ receptors in airway reactivity [93]. Moreover, in a model of experimental non-atopic asthma in mice, in vivo intranasal treatment with the CB_1_ selective agonist, ACEA was shown to prevent airway hyperreactivity [111].

Inhaled AEA pre-treatment of guinea pigs has shown to prevent leukotriene-D_4_ aerosol-induced bronchospasm [114], an effect suggesting the role of endocannabinoid targeted therapy in airway diseases. AEA has also been shown to reduce trans-epithelial resistance in airway cells, which indicates an increase in barrier permeability [119]. However, this effect seems to be mediated by the metabolism of AEA to one or more LOX and COX metabolites rather than CB_1_ and CB_2_ receptor-dependent mechanisms. Increased AEA levels in asthmatic patients may contribute to increased permeability of the epithelium through degradation to arachidonic acid metabolites. Therefore, preventing AEA hydrolysis in the airways may help to prevent epithelial permeability in asthma [119]. A strategy for this and to increase AEA tissue levels is to inhibit the activity of the FAAH enzyme, which is responsible for the hydrolysis of AEA and other related amidated signaling lipids, such as PEA, N-oleoylethanolamide (OEA) and linoleoylethanolamide (LEA). Inhibition of FAAH has been shown to produce antitussive effects in guinea pigs [120]. This effect was suggested to be mediated by elevated fatty amino acids, which act on cannabinoid (CB_2_) receptors on vagal sensory nerves [120]. Therefore, increasing the endocannabinoid levels by FAAH inhibition can be a promising strategy as a new treatment option for antitussive therapy.

The effect of endocannabinoids was studied in the LPS-induced experimental acute lung injury (ALI) model in mice [121]. It is well known that ALI may occur due to sepsis, pneumonia, acid aspiration, toxic inhalation, etc. In their study, Costola-de-Souza et al. showed that treatment with the MAGL inhibitor, JZL184 attenuated the pathological changes of ALI by increasing 2-AG levels in the lungs. However, in their study, neither CB_1_ nor CB_2_ receptor antagonists were able to fully block the effect of JZL184, suggesting the involvement of other mechanisms such as stimulation of non-cannabinoid receptors or an increase in non-cannabinoid products like prostaglandins. Abohalaka et al. studied the effects of FAAH or MAGL inhibitor treatments in LPS-induced airway inflammation and airway hyperreactivity [109]. These inhibitors were applied either systemically (i.p. route) or locally (i.n.) before LPS administration to mice. Both FAAH inhibitor and MAGL inhibitor treatments were found to be effective in preventing airway hyperreactivity, whether they were applied via the i.p. or i.n. route. The treatments were also able to prevent the histopathological changes in the lungs, except the local i.n. application of the FAAH inhibitor URB597 [109]. These effects were possibly mediated by the increased AEA and 2-AG levels in the lungs (unpublished data). These data suggest that increasing the concentration of endocannabinoids in the airways by the inhibition of primary endocannabinoid degrading enzymes FAAH and MAGL can prevent airway hyperreactivity and airway inflammation. However, more studies should be performed in order to clarify the effects of FAAH and MAGL inhibitors in the airways since different fatty amino acids are also substrates for these enzymes.

The endocannabinoid system also contributes to pulmonary fibrosis, which is a life-threatening disease. Endocannabinoids are shown to promote the progression of fibrosis in liver [122,123,124], kidney [125,126], heart [127], and skin [128]. Studies in mice suggest that CB_1_ receptors are associated with radiation-induced pulmonary fibrosis [129]. Cinar et al. performed a detailed study in order to identify the contribution of CB_1_ receptors in lung fibrosis, and have shown that both the genetic deletion of CB_1_ receptors or their pharmacological inhibition with a CB_1_ antagonist can attenuate lung inflammation and fibrosis, and hence increase animal survival in a mouse model of radiation-induced pulmonary fibrosis [130]. This finding indicates a different pattern than other studies in which cannabinoid CB_1_ receptor stimulation has been raised as a therapeutic approach, as discussed above. This controversy may be due to the models used to induce fibrosis and related pathophysiology of fibrosis, which is different from acute or allergic airway inflammation. However, although CB_1_ activation can prevent airway hyperreactivity and inflammation, CB_2_ receptor antagonism has also been suggested as a therapeutic strategy for allergic diseases [78,118].

## 4. Conclusions

The studies about the contribution of the endocannabinoid system in the airways indicate the importance of both CB_1_ and CB_2_ receptors (Table 1). Among these two receptors, CB_1_ subtype is more likely to be involved in the functional reactivity of the airways as its stimulation can inhibit the contraction of airway smooth muscle. This effect seems to be mediated by the inhibition of acetylcholine release from cholinergic nerves, rather than a direct effect on the smooth muscle itself. Unlike CB_1_ receptors, CB_2_ receptors are likely to be involved in the mechanisms for neurogenic inflammation, probably acting through the sensory nerves (Figure 1). The contribution of both receptors in the immune modulation of airways is well established, as discussed above. The data of the present literature indicate a significant contribution of CB_2_ receptors in allergic diseases, which can be considered for the treatment of allergic asthma. However, the possible involvement of CB_1_ receptors should not be excluded, since they are expressed and functional almost in every immune cell. Therefore, appropriate cannabinoid receptor ligands may be rational candidates for the treatment of airway diseases because of their anti-inflammatory and bronchodilatory effects.

Altering the tissue levels of endocannabinoids as a therapeutic strategy is a complex issue. One reason for that is cannabinoids have other targets then CB receptors, which can cause unpredictable effects. The enzymes responsible for endocannabinoid metabolism also have other bioactive by-products, which have significant biological effects. Furthermore, in addition to FAAH or MAGL, other enzymes are also involved in endocannabinoid metabolism, such as COX-2, which leads to the production of various inflammatory prostaglandins. In this respect, the elevation of AEA or 2-AG tissue levels by FAAH or MAGL inhibition were shown to enhance their COX-2-mediated oxidation, and increase prostamide and PG-G signaling [131,132]. These factors make the therapeutic use of FAAH and MAGL inhibitors more complicated, and may partly account for the failure of some FAAH inhibitors like PF-04457845, BIA 10-2474, PF-06818883, V-158866 in clinical trials [6,133,134,135]. For instance, the reasons for the interruption of the clinical trial of BIA 10–2474 due to a death of a volunteer are still not fully established and thought to be related to the off-target effects of FAAH inhibitors [134,135]. However, elevated endocannabinoid levels were not reported to be associated with severe toxic effects on the central nervous system [134]. The specificity of the molecule itself or its metabolite(s) should be considered for the off-target effects, which can cause unpredictable adverse events in clinical trials. Therefore, more studies should be performed in order to clarify the contribution of by-products and other bioactive fatty acids in airway inflammation.

## Figures and Tables

**Table 1:** Functional cannabinoid (CB) receptors in the airways and immune cells.

Cell Type	Receptor	Species and Used Cells/Tissues in Related Studies
Alveolar Type-II epithelial cells	CB_1_	Human [130]
Nerves	CB_1_	Rat, Human [89,92]
Macrophages	CB_1_/CB_2_	Human CD68(+), CD36(+) macrophages (CD: cluster of differentiation); THP-1 human monocytic cell line-derived macrophages; Murine RAW264.7 macrophage cell line [73] Human lung macrophages; lung cancer-associated macrophages [136] Human monocyte-derived macrophages [137]
Mast cells	CB_1_/CB_2_	RBL2H3 mast cell line [58] Human CTS-mast cells [59] Human mucosal type mast cells [60]
Eosinophils	CB_1_/CB_2_	EoL-1 cells [69] Human eosinophils from peripheral blood [69,70,71,138]
Dendritic cells	CB_1_/CB_2_	Mouse bone marrow-derived dendritic cells [49,80] Human dendritic cells from peripheral blood [79]
Neutrophils	CB_1_/CB_2_	Human promyelocytic cell line HL60 [33,81] Human neutrophils from peripheral blood [33,69,70,71,81]
B cells	CB_1_/CB_2_	Human B cells from peripheral blood [33,54,74] Human tonsillar B cells [54] Human B lymphoblastoid cell line DAUDI [33]
T lymphocytes	CB_1_/CB_2_	Human T cells from peripheral blood [74,139]
Basophils	CB_1_/CB_2_	Human basophils from peripheral blood [138]
NK cells	CB_1_/CB_2_	Human NK cells from peripheral blood [33]
Monocytes	CB_1_/CB_2_	Human monocytes from peripheral blood [33,72,73,74] Human monocytic cell line U937 [33]
